# Comparative evaluation of bipolar versus monopolar energy platforms in G-POEM for gastroparesis: technical performance, learning curves, and clinical outcomes

**DOI:** 10.1007/s00464-026-12746-0

**Published:** 2026-03-19

**Authors:** Inanc S. Sarici, Himsikhar Khataniar, Jennifer M. Kolb, Sven E. Eriksson, Kirsten Newhams, Katherine Albus, Ping Zheng, Shahin Ayazi

**Affiliations:** 1 Chevalier Jackson Esophageal Research Center, Western Pennsylvania Hospital, Allegheny Health Network, 4815 Liberty Avenue, Suite 439, Pittsburgh, PA 15224 USA; 2 Foregut Division, Surgical Institute, Allegheny Health Network, Pittsburgh, PA USA; 3 Department of Medicine, Allegheny Health Network, Pittsburgh, PA USA; 4 Vatche and Tamar Manoukian Division of Digestive Diseases, David Geffen School of Medicine at UCLA, Greater Los Angeles VA Healthcare System, Los Angeles, CA USA; 5 Department of Surgery, Drexel University, Philadelphia, PA USA

**Keywords:** Gastroparesis, G-POEM, Speedboat knife, Triangle-tip knife, Gastroparesis Cardinal Symptom Index (GCSI), Gastric emptying scintigraphy

## Abstract

**Background:**

Gastric peroral endoscopic pyloromyotomy (G-POEM) is an established treatment for medically refractory gastroparesis, traditionally performed using monopolar electrosurgical platforms. A novel bipolar energy device integrates cutting and coagulation into a single instrument and may streamline submucosal tunneling procedures. While feasibility has been demonstrated, direct comparisons between bipolar and monopolar energy platforms are lacking. This study aimed to compare technical performance, learning curves, perioperative metrics, and clinical outcomes between these two platforms in G-POEM.

**Methods:**

Patients undergoing G-POEM using either a bipolar energy platform (Speedboat knife, SBK) or a monopolar energy platform (Triangle-tip knife, TTK) between 2019 and 2024 were retrospectively reviewed. Primary outcomes included technical success, operative time, and learning curve inflection points based on operative efficiency. Secondary outcomes included perioperative complications, symptom resolution, postoperative Gastroparesis Cardinal Symptom Index (GCSI) scores, and gastric emptying scintigraphy results.

**Results:**

A total of 111 patients were included (SBK: 23; TTK: 88). Baseline characteristics were similar between groups. Technical success was achieved in 95.7% of SBK and 100% of TTK cases (*p* = 0.207). One SBK case required conversion to TTK. Learning curve analysis showed inflection points at 7 procedures for SBK and 8 for TTK, based on operative time reduction (SBK: 98.0 vs 60.1 min, *p* = 0.001; TTK: 83.3 vs 63.1 min, *p* = 0.005). Median operative time, hospital stay, and 30- and 90-day readmission rates were comparable between groups (all *p* > 0.05). No leaks or perforations occurred. At a mean follow-up of 13.4 (10) months, there were no differences in predominant symptom resolution (*p* = 1.000), postoperative GCSI scores (*p* = 0.985), or GCSI subdomain scores (all *p* > 0.05). Improvement in gastric emptying scintigraphy was also similar (*p* = 0.410). Follow-up duration was comparable (*p* = 0.303).

**Conclusion:**

Bipolar energy platforms demonstrated comparable technical success, learning curves, and clinical outcomes to monopolar energy platforms for G-POEM. Bipolar energy may serve as a safe and effective alternative for endoscopic pyloromyotomy in patients with medically refractory gastroparesis.

**Graphical abstract:**

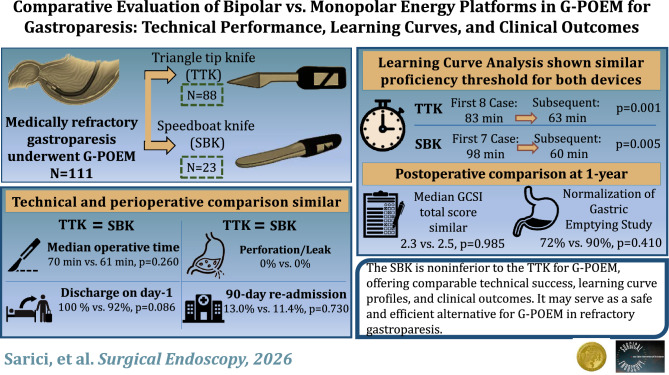

Gastroparesis is a motility disorder characterized by delayed gastric emptying in the absence of mechanical obstruction. It affects approximately 10 to 40 per 100,000 individuals in the USA and is associated with increasing healthcare utilization [1]. The most common etiologies include diabetic and idiopathic causes, though post-surgical and medication-induced forms are also recognized [2]. First-line management typically involves dietary modification and prokinetic agents; however, up to 30–50% of patients remain symptomatic despite optimized medical therapy and are considered to have medically refractory gastroparesis, ultimately requiring procedural intervention [3–9].

Gastric peroral endoscopic pyloromyotomy (G-POEM) is a well-established therapeutic intervention for patients with medically refractory gastroparesis [10]. The procedure aims to relieve pyloric outflow obstruction by performing a targeted myotomy of the pyloric sphincter through a submucosal tunneling approach, thereby improving gastric emptying and alleviating symptoms. Traditionally, G‑POEM is performed using a monopolar electrosurgical knife, most commonly the triangle tip knife (TTK), which allows for precise mucosal incision and controlled myotomy. Reported clinical success rates with TTK range from 68 to 80% in large multicenter studies and systematic reviews, with durable symptom improvement observed in up to 70% of patients [11, 12]. Recently, alternative energy platforms have been explored to improve procedural efficiency. The speedboat knife (SBK) is a novel bipolar endoscopic device that integrates submucosal injection, radiofrequency (RF) cutting, and microwave coagulation in a single instrument, potentially reducing the need for instrument exchanges during tunneling and myotomy. Although relatively new, the SBK has been utilized in various endoscopic procedures and, more recently, in G-POEM, with preliminary case series suggesting that it is a safe and feasible technique [13–15]. These studies suggest that the SBK platform may offer improved safety through more controlled dissection, reduced collateral thermal injury, and increased procedural efficiency by limiting device exchanges [14–17]. Such advantages could be particularly beneficial in submucosal tunneling procedures like G-POEM.

Despite growing adoption of G-POEM, most available outcomes data are based on procedures performed using a monopolar platform (TTK). Although the bipolar platform (SBK) may offer technical advantages, direct comparative studies are lacking. Furthermore, little is known about how these two platforms differ in terms of procedural learning curves. Therefore, the aim of this study was to compare the technical performance, perioperative outcomes, clinical efficacy, and learning curves of SBK versus TTK in patients undergoing G-POEM for medically refractory gastroparesis.

## Materials and methods

### Study population

This is a retrospective review of prospectively collected data on patients who underwent G-POEM for the management of medically refractory gastroparesis at Allegheny Health Network hospitals (Pittsburgh, PA) between 2019 and 2024. Patients with at least 1 year of postoperative follow-up were included. Exclusion criteria were age under 18 years, prior pyloric drainage procedures, or anatomic abnormalities such as mechanical gastric outlet obstruction. This study was approved by the Institutional Review Board of Allegheny Health Network (IRB #2020–076).

### Gastroparesis diagnosis and preoperative assessment

Gastroparesis was diagnosed based on a comprehensive foregut evaluation incorporating both symptom characterization and objective diagnostic testing. All patients underwent a standardized workup that included gastric emptying scintigraphy (GES), completion of the Gastroparesis Cardinal Symptom Index (GCSI), and classification of disease etiology as diabetic, idiopathic, or postsurgical. Predominant symptoms such as nausea/vomiting, bloating, or early satiety were documented. Alternative diagnoses, including rumination syndrome, functional dyspepsia, superior mesenteric artery syndrome, and eating disorders, were excluded through clinical history, physical examination, and ancillary testing. Patients diagnosed with gastroparesis were initially managed with dietary modification and prokinetic therapy. Only those with persistent symptoms despite optimized medical management were considered for G-POEM.

### GCSI assessment

The GCSI is a validated patient-reported outcome measure assessing nine gastroparesis-related symptoms on a Likert scale from 0 (none) to 5 (very severe). The nausea/vomiting subscore reflects responses to nausea, retching, and vomiting; the early satiety subscore includes stomach fullness, inability to complete a normal meal, excessive fullness, and loss of appetite; and the bloating subscore includes bloating and visible distention. The total GCSI score was calculated as the mean of these three sub-scores, while the summed total score represented the aggregate of all nine symptom ratings. Both the predominant symptom and GCSI scores were recorded preoperatively and at postoperative follow-up.

### Gastric emptying scintigraphy technique and interpretation

Gastric emptying scintigraphy (GES) was performed using a standardized radiolabeled solid meal containing 1 mCi of technetium 99 m sulfur colloid. Anterior and posterior planar images of the abdomen were obtained for 60 s immediately after ingestion and at hourly intervals over a 4-h period. The gastric region of interest was delineated, and radiotracer counts were measured at each time point. Attenuation-corrected counts were compared with baseline values to determine the percentage of meal retention. Delayed gastric emptying was defined as > 10% retention at 4 h, in accordance with established guidelines.

### G‑POEM technique

All G-POEM procedures were performed under general anesthesia by a small group of high-volume foregut surgeons using a standardized technique. All attending surgeons had substantial prior experience with advanced endoscopic foregut interventions, including both esophageal POEM and G-POEM, and were proficient in submucosal tunneling and endoscopic myotomy techniques prior to adoption of the SBK. Clinical fellows were actively involved in all procedures. A submucosal cushion was created approximately 2–3 cm proximal to the pylorus along the lesser curvature using a saline and dye solution. A transverse mucosal incision was then made to initiate submucosal tunneling.

### SBK group

**Fig. 1:**
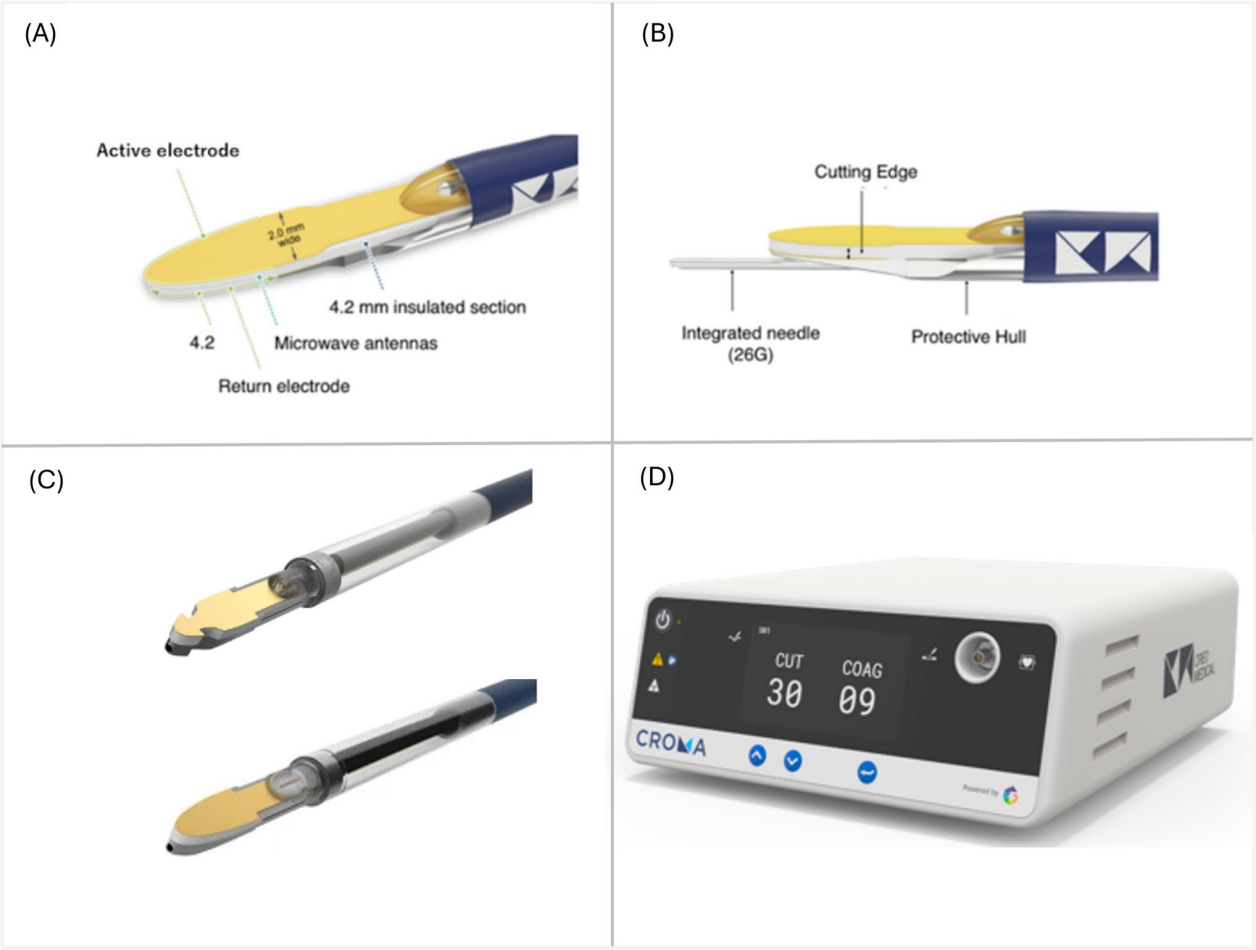
Design and functionality of the SBK system. **A** Schematic view of the distal end of an earlier-generation SBK illustrating the active bipolar cutting electrode, return electrode, integrated microwave antennas, and insulated shaft. **B** Lateral schematic view demonstrating the cutting edge, integrated injection needle, and protective hull designed to facilitate controlled submucosal dissection. **C** Representative images of two low-profile SBK variants compatible with diagnostic endoscopes. **D** The CROMA electrosurgical generator used to deliver bipolar radiofrequency energy for tissue cutting and microwave energy for coagulation during endoscopic procedures. Panels A and B are adapted from an open-access publication (*New Kid on the Block: “Speedboat,”* Journal of Digestive Endoscopy, 2022; CC BY-NC-ND 4.0), and panels C and D were provided courtesy of the manufacturer

The SBK (Speedboat-RS2; Creo Medical Ltd., UK) is a novel bipolar endoscopic dissection tool specifically designed for submucosal tunneling and myotomy procedures. Compatible with a 3.7-mm working channel of a single-channel therapeutic endoscope, the device features a fully rotatable shaft, front and lateral cutting edges, and an insulated upper surface to allow precise dissection while minimizing thermal injury to underlying tissue. Its integrated 26-gauge needle allows for real-time submucosal injection and irrigation. The SBK delivers bipolar radiofrequency energy (400 kHz, 35 W) for cutting and microwave energy (5.8 GHz, 10 W) for coagulation, eliminating the need for instrument exchange during the procedure (Fig. 1). A newer, smaller profile version of the SBK is compatible with diagnostic endoscopes with a working channel ≥ 2.7 mm. This lower-profile device was not used in the present cohort.

During the procedure, the tip of the SBK was used to initiate a longitudinal mucosal incision, approximately 4 cm in length, along the lesser curvature. This was followed by downhill tunneling toward the pylorus, facilitated by the device’s rotatable body and protective hull, which provided both stability and depth control to avoid unintentional muscle injury. The full-thickness myotomy began just distal to the pyloric ring and was extended proximally into the antrum by approximately 1.5 cm. The bipolar blade enabled both forward and lateral muscle division, while microwave coagulation was applied selectively to submucosal vessels and for hemostasis throughout the dissection.

### TTK group

In the TTK group, the triangle-tip knife (Olympus, Tokyo, Japan) was used to perform the mucosotomy, submucosal tunneling, and pyloromyotomy. The TTK is a needle-type electrosurgical knife compatible with standard diagnostic endoscopes with a ≥ 2.7-mm working channel. The triangle-tip design enabled controlled and focused energy delivery for muscle division while minimizing mucosal injury. The TTK is a needle-type electrosurgical knife with a 4.5 mm cutting length, a 0.4 mm shaft diameter, and a triangular tip measuring 0.7 mm in radius and 0.4 mm in thickness. After mucosotomy, submucosal tunneling was carried out using TTK to reach the level of the pyloric ring. A selective full-thickness myotomy of the circular pyloric muscle fibers was then performed under direct endoscopic visualization using the same device.

In both groups, after completing the myotomy, the submucosal tunnel was irrigated, and the mucosal entry site was carefully inspected for serosal injury before being closed with endoscopic clips (Resolution™, Boston Scientific, Marlborough, MA). All patients underwent an upper gastrointestinal contrast study on postoperative day one to evaluate for contrast extravasation or obstruction.

### Learning curve analysis

To assess the learning curve and identify the turning point where a significant improvement in operative efficiency occurred, we applied a change-point analysis based on operative time. First, procedures were ordered chronologically by date. Each procedure date was evaluated as a potential cut point to divide the series into two phases: an early phase (prior to the cut point) and a late phase (after the cut point). For each potential cut point, mean operative times between the two phases were compared using Student’s *t* test. The turning point was defined as the procedure date associated with the highest absolute *t* value, indicating the greatest difference in operative duration between early and late phases. The corresponding case number at this turning point was recorded as the estimated learning threshold. To visually represent the analysis, a graph was generated plotting the absolute *t* values against the cumulative case number, allowing for intuitive identification of the learning curve inflection point. Since procedures were performed by multiple surgeons within a training program, with fellows assisting or performing portions under supervision, the analysis reflects a center-wide learning pattern rather than that of individual operators.

### Outcomes and statistical analysis

Baseline demographic, procedural, and postoperative outcomes were compared between patients who underwent G-POEM using the TTK or the SBK for medically refractory gastroparesis. The primary outcome was technical success, defined as completion of the procedure without switching to an alternative knife. Secondary outcomes included operative efficiency (operative time and number of closure clips), perioperative and postoperative complications (perforation, pneumoperitoneum, and leak), and clinical outcomes including resolution of predominant symptoms, postoperative GCSI scores, 30- and 90-day length of hospital stay, and improvement in gastric emptying scintigraphy results. Preoperative and postoperative gastric emptying scintigraphy results were evaluated to assess improvement in gastric retention and normalization of gastric emptying. Values for continuous variables are presented as mean (standard deviation) or median with interquartile range, as appropriate. Categorical variables are reported as frequency and percentage. Comparisons between groups were performed using nonparametric tests, including the Mann–Whitney *U* test for continuous variables and Pearson’s chi-square test or Fisher’s exact test for categorical variables, as appropriate. A *p* value of < 0.05 was considered statistically significant. All statistical analyses were conducted using SAS software (version 9.4, SAS Institute, Cary, NC).

## Results

### Patient characteristics

A total of 111 patients underwent G-POEM during the study period, including 23 in the SBK group and 88 in the TTK group. The mean (SD) age of the cohort was 51.6 (15.4) years, and 80.2% were female with a mean (SD) BMI of 29.9 (7.7) kg/m^2^. Gastroparesis etiology was idiopathic in 61.3%, diabetic in 27.0%, and post-surgical in 11.7%. The most predominant symptom was nausea/vomiting (58.6%), followed by bloating (28.8%) and abdominal pain (9.9%). There were no statistically significant differences in demographic variables, BMI, etiology, or symptom distribution between the SBK and TTK groups as shown in Table 1.

**Table 1:** Comparison of the demographic and baseline characteristics between speedboat knife (SBK) and triangle-tip knife (TTK) patients

Characteristics	SBK (*N* = 23)	TTK (*N* = 88)	*p* value
Age, median (IQR)	61 (38–70)	52 (44–60)	0.2896
Sex (female), *N* (%)	16 (69.6%)	73 (83.0%)	0.2375
BMI, median (IQR)	28.5 (24.7–33.3)	28.3 (25.0–33.9)	0.9768
Gastroparesis type, *N* (%)			
Diabetic	10 (43.5%)	20 (22.7%)	0.126
Idiopathic	12 (52.2%)	56 (63.6%)	
Postsurgical	1 (4.3%)	12 (13.6%)	
GCSI, median (IQR)			
Total score	3.1 (2.9–3.8)	3.1 (2.0–3.9)	0.5240
Nausea/vomiting score	2.3 (1.7–3.7)	2.7 (0.7–4.0)	0.9177
Early satiety score	3.4 (2.8–4.0)	3.3 (2.0–4.3)	0.9939
Bloating score	4.0 (3.0–5.0)	3.5 (2.0–4.0)	0.1748
Predominant symptom, *N* (%)			
Nausea/vomiting	16 (69.6%)	49 (55.7%)	0.279
Bloating	5 (21.7%)	27 (30.7%)	
Abdominal pain	1 (4.3%)	10 (11.4%)	
Early satiety	0 (0.0%)	2 (2.3%)	
Other	1 (4.3%)	0 (0.0%)	
GES % 4-h retention, median (IQR)	24.0 (14.0–63.0)	27.7 (14.0–46.0)	0.6194

### Technical, procedural, and safety outcomes

Technical success was achieved in 95.7% of the SBK group and 100% of the TTK group (*p* = 0.207). One case in the SBK group required intra-procedural conversion to TTK because of a technical limitation encountered during the procedure, after which the case was completed successfully without complication. There were no differences between the SBK and TTK groups in mean operative time, mucosotomy length, and the number of clips required for mucosal closure, as shown in Table 2. There were no perforations or leaks in either group. Pneumoperitoneum occurred in 3.4% of cases in the TTK group, none of which required intervention, while no cases were observed in the SBK group (*p* = 1.000).

**Table 2:** Comparison of technical factors and perioperative characteristics between speedboat knife (SBK) and triangle-tip knife (TTK) patients

Characteristics	SBK (*N* = 23)	TTK (*N* = 88)	*p* value
Operative time (min), median (IQR)	70 (55–86)	61 (50–76)	0.2606
Mucosotomy length, (cm), median (IQR)	2.3 (2.0–2.9)	2.6 (2.1–3.2)	0.7304
Closure clips, median (IQR)	5 (5–10)	6 (5–7)	0.6675
Perforation/leak, *N* (%)	0 (0%)	0 (0%)	–
Pneumoperitoneum, *N* (%)	0 (0%)	3 (3.4%)	1.0000
LOS (days), median (IQR)	1 (1–1)	1 (1–1)	0.0696
30-day readmission, *N* (%)	2 (8.7%)	8 (9.1%)	1.0000
90-day readmission, *N* (%)	3 (13.0%)	10 (11.4%)	0.7304

The majority of patients in both groups were discharged on postoperative day one, and the median hospital length of stay was similar between groups. No Clavien–Dindo grade III or higher complications were observed in either group, and there were no significant differences in 30-day or 90-day readmission rates between the groups, as shown in Table 2.

### Learning curve comparison

**Fig. 2:**
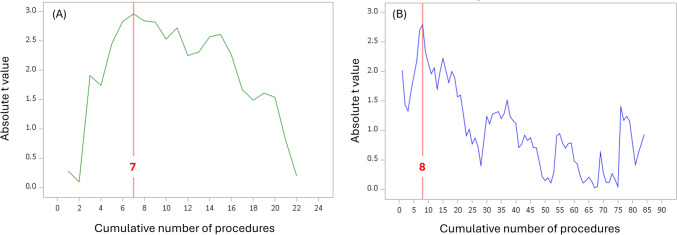
Learning curve analysis for G-POEM using SBK and TTK. **A** In the SBK group, the highest absolute *t* value (2.96) was observed after the 7th procedure, indicating the proficiency threshold. **B** In the TTK group, the learning curve peaked at the 8th procedure (*t* value: 2.79). These inflection points were determined using cumulative *t* value analysis, with red vertical lines indicating proficiency thresholds (Color figure online)

Learning curve analysis demonstrated similar proficiency thresholds for both devices. In the SBK group, the highest absolute *t* value (2.96) occurred after the 7th case, with operative time decreasing from 98.0 to 60.1 min (*p* = 0.001; Fig. 2A, Table 3). In the TTK group, the peak *t* value (2.79) was observed after the 8th case, with a corresponding drop in operative time from 83.3 to 63.1 min (*p* = 0.005; Fig. 2B, Table 3). These findings suggest comparable learning curves between SBK and TTK. In addition to the center-wide change-point analysis, a review of operative times by individual surgeons demonstrated a consistent qualitative trend toward reduced operative duration with increasing procedural experience, although the sample size was insufficient for formal surgeon-level modeling.

**Table 3:** Comparison of operative time between speedboat knife (SBK) and triangle-tip knife (TTK) groups based on learning curve analysis

Device group	Case subset	Operative time (min)	*p* value
		Mean (SD)	
Speedboat knife (SBK)	First 7 cases	98.0 (31.0)	0.001
	Remaining 16 cases	60.1 (17.8)	
Triangle-tip knife (TTK)	First 8 cases	83.3 (13.0)	0.005
	Remaining 77 cases	63.1 (19.1)	

### Clinical and symptom outcomes

At a mean (SD) follow-up of 13.4 (10) months after surgery, predominant symptom resolution was achieved in 65.2% of SBK patients and 65.9% of TTK patients (*p* = 1.000). There were no significant differences between the two groups in postoperative GCSI total scores or in any of the sub-scores for nausea/vomiting, early satiety, and bloating, as shown in Table 4. Postoperative improvement in gastric emptying, based on 4-h retention on scintigraphy, was observed in 90.0% of patients in the SBK group and 72.2% in the TTK group (*p* = 0.4103).

**Table 4:** Comparison of postoperative outcomes between speedboat knife (SBK) and triangle-tip knife (TTK) patients

Characteristics	SBK (*N* = 23)	TTK (*N* = 88)	*p* value
Predominant symptom resolution, *N* (%)	15 (65.2%)	56 (65.9%)	1.0000
GCSI, median (IQR)			
Total score	2.3 (1.2–3.8)	2.5 (1.4–3.3)	0.9850
Nausea/vomiting score	2.0 (0.3–3.0)	1.7 (0.3–2.7)	0.7764
Early satiety score	3.3 (2.3–4.3)	2.5 (1.5–3.8)	0.1995
Bloating score	1.0 (1.0–3.5)	3.0 (1.5–5.0)	0.2309
GES % 4-h retention, median (IQR)	9 (1–41)	13 (5–32)	0.5919

## Discussion

Gastric peroral endoscopic pyloromyotomy (G-POEM) using the triangle-tip knife is a well-established and effective treatment for medically refractory gastroparesis, with reported clinical success rates ranging from 68 to 90% and durable symptom relief [18, 19]. A retrospective single-center study of 115 patients undergoing TTK-based G-POEM reported a 100% technical success rate [20], supporting the procedural reliability of this approach. The SBK, a newer bipolar energy platform, was originally developed for endoscopic submucosal dissection, with its feasibility first demonstrated by Saunders et al. in a porcine model in 2013 [16]. Human applications were first reported in colorectal ESD, followed by successful use in submucosal tunneling procedures, including esophageal POEM [17]. In a multicenter retrospective study involving 17 patients, SBK achieved 100% technical success in esophageal POEM, with 76.4% of cases completed without requiring any additional instruments [21]. More recently, Khirfan et al. reported the first use of SBK in G-POEM for gastroparesis, demonstrating symptomatic improvement and GCSI score reduction from 35 to 17 at 4-week post-procedure. In our study, technical success was achieved in 95.7% of SBK cases and 100% of TTK cases, reflecting a high success rate for both devices [13]. This study fills a gap in the literature by providing the first comparative evaluation of SBK and TTK techniques in G-POEM. Our findings indicate that SBK offers clinical efficacy comparable to TTK, with similar postoperative symptom scores and rates of symptom resolution. Notably, the learning curves for both devices were also similar, with early procedural efficiency achieved after a limited number of cases. These results support SBK as a safe, effective, and technically feasible alternative to conventional monopolar techniques in the treatment of gastroparesis. While prior studies have suggested potential safety or workflow advantages of SBK, our comparative analysis demonstrated equivalent clinical, technical, and safety outcomes between SBK and TTK. These findings support SBK as an efficient alternative energy platform rather than a superior one.

Complications reported across G-POEM studies have included bleeding, perforation, capnoperitoneum, and abscess formation at the mucosotomy site, with varying degrees of severity [18]. A recent systematic review and meta-analysis of 20 studies involving 797 patients reported an overall adverse event rate of 10.92%, underscoring the importance of procedural safety [22]. In our study, no significant intraoperative complications such as mucosal perforation or serosal injury were observed in either group. Pneumoperitoneum occurred in three patients (3.4%) in the TTK group but was self-limiting and required no intervention, while no cases were noted in the SBK group. A retrospective multicenter study of 217 patients undergoing TTK-based G-POEM similarly reported a rate of 1.8% [12]. The SBK incorporates several features aimed at enhancing procedural safety. It is designed with three functional edges on the tip and both sides to facilitate precise mucosal incision and submucosal dissection. The device includes bipolar cutting blades positioned laterally, with radiofrequency energy delivered from the top blade and returned via the bottom blade, enabling focused energy application while minimizing unintended thermal spread. A key safety feature is the protective hull at the base of the device, which helps maintain a safe distance between the energy source and the muscle layer, reducing the risk of perforation and improving stability during dissection [23]. In a porcine model comparing colonic ESD using the SBK and a conventional monopolar device, SBK was associated with less muscle injury and a reduced need for endoscopic clipping [24]. Furthermore, comparative animal studies have shown that microwave coagulation, as utilized in the SBK, results in fewer histologic alterations to the muscle layer compared to monopolar and conventional bipolar devices, further supporting its potential safety advantage [25]. While our findings showed no pneumoperitoneum in the SBK group, a recent report noted capnoperitoneum requiring needle decompression and retroperitoneal CO_2_ accumulation requiring temporary procedural pause in two patients undergoing esophageal POEM with SBK [17]. These patients had type II and III achalasia and required extended myotomy durations exceeding 60 min, suggesting that longer operative times may increase the risk of insufflation-related complications regardless of the device used.

Normalization in gastric emptying studies remains the only objective marker of improved gastric motility in patients undergoing G-POEM. A single-center study involving 115 patients who underwent G-POEM with the TTK demonstrated a significant reduction in mean (SD) half-emptying time from 49.15 (36.29) min to 13.11 (8.92) min at 6-month post-procedure, with this improvement shown to be durable up to 5 years [20]. Although no prior studies have evaluated gastric emptying outcomes after G-POEM using the SBK, our study demonstrated comparable normalization rates, with 90.0% of SBK patients and 72.2% of TTK patients showing improvement on GES. These outcomes are consistent with previous literature, all of which have involved the TTK platform; a meta-analysis reported GES normalization rates ranging from 68 to 80%, and a retrospective single-center trial by Gonzalez et al. observed a 75% normalization rate using TTK [18, 26, 27]. Collectively, these findings support the use of either dissection platform for effective enhancement of gastric emptying following G-POEM.

G-POEM using the triangle tip knife has been consistently associated with effective outcomes, with large series reporting mean operative times ranging from 68 to 84 min [28]. The SBK, a multifunctional bipolar platform, delivers radiofrequency energy for cutting, microwave energy for coagulation, and features an integrated injection needle. This design enables mucosal incision, submucosal tunneling, injection, dissection, and hemostasis to be performed using a single instrument, theoretically reducing the need for accessory exchanges and thereby improving procedural efficiency. While limited data are available on SBK use specifically in G-POEM, prior reports in various endoscopic interventions have shown operative times ranging from 25 to 90 min, depending on procedure type, lesion size, and location [15, 17, 29, 30]. For instance, a single-center retrospective study involving 64 patients with proximal rectal polyps (median size 4 cm) reported a median resection time of 90 min (range 15–270 min) [31]. Simsek et al. reported a mean operative time of 126 min (range 45–219 min) for esophageal POEM using SBK in 17 achalasia patients, with an average submucosal tunnel length of 12.1 cm [21]. Another study involving 10 submucosal tunneling cases, including seven POEM procedures, reported a mean duration of 59.1 ± 31.1 min [17]. In our study, mean operative times were comparable between SBK (71.7 min) and TTK (65.2 min), despite the potential time-saving advantage of the all-in-one SBK platform. Importantly, we did not observe objective differences in procedural efficiency between the two platforms, and metrics such as device exchange frequency, accessory utilization, and number of injections were not specifically measured in this study. Therefore, any potential efficiency or workflow advantages of SBK should be interpreted as conceptual and based on prior literature, rather than as demonstrated superiority over TTK in our cohort.

One of the aims of this study was to compare the learning curves associated with two techniques for performing G-POEM. Using operative time as a surrogate for technical proficiency, we identified the inflection point for each approach—defined as the case number with the most statistically significant reduction in procedure duration. Both techniques demonstrated relatively short learning curves. The SBK group reached its turning point after 7 cases, while the TT knife group reached it after 8. This minimal difference suggests that device selection does not substantially influence the rate of skill acquisition. In addition, Triangle Tip Knife was the standard device at our center prior to the introduction of the SBK platform in late 2021. As such, the SBK cohort represents a later phase in our institutional G-POEM experience, during which surgeons had already accumulated experience with the TTK. This sequence may have contributed to the relatively rapid proficiency observed with SBK. These findings support the feasibility of both approaches for clinical adoption, particularly by endoscopists with prior experience in submucosal dissection. Our results are consistent with existing literature indicating that G-POEM has a favorable learning curve compared to other advanced endoscopic procedures, with proficiency typically achieved after 7–10 cases. This further supports the notion that learning efficiency is driven more by operator experience and case volume than by the choice of dissection platform. Although the SBK integrates cutting, injection, and coagulation into a single device—potentially streamlining the procedure—our findings suggest that this added functionality does not significantly reduce the learning curve.

Several important limitations should be acknowledged. This was a retrospective, single-center study, which may limit the generalizability of our findings and introduce potential selection bias. The sample size, particularly for the SBK group, was relatively small and not powered to detect subtle differences in clinical or procedural outcomes. Accordingly, the absence of statistically significant differences should be interpreted as a lack of detected difference rather than evidence of equivalence between SBK and TTK. Because SBK was introduced later in the study period, time-trend bias related to cumulative institutional experience cannot be excluded. In addition, the limited number of SBK cases and their distribution among a small group of surgeons precluded surgeon-level learning curve analysis; therefore, learning curves reflect center-level adoption rather than individual operator performance. Future prospective studies are needed to address these limitations and more comprehensively compare the clinical and economic implications of TTK and SBK in G-POEM.

## Conclusion

The SBK appears to be a safe and effective alternative to the TTK for performing G-POEM in patients with medically refractory gastroparesis. It demonstrates comparable technical performance, operative times, and postoperative outcomes, including symptom resolution and improvement in gastric emptying. With increased familiarity and broader adoption, SBK may offer additional procedural advantages by reducing instrument exchanges and enhancing technical efficiency. Prospective multicenter studies with long-term follow-up are warranted to confirm these findings and assess cost-effectiveness.
